# The Surgical Treatment of Puerperal Peritonitis

**Published:** 1892-07

**Authors:** W. E. B. Davis

**Affiliations:** President of the Tri-State Medical Society of Alabama, Georgia and Tennessee; Secretary of the Southern Surgical and Gynecological Association, etc. Rome, Georgia


ATLANTA
MEDICAL AND SURGICAL JOURNAL.

VOL. IX. JULY, 1892. No. 5.

EDITED BY
LUTHER B. GRANDY, M. D., AND MILLER B. HUTCHINS, M. D.,
WITH THE CO-OPERATION OF
H. V. M. MILLER, M. D., LL. D., VIRGIL O. HARDON, M. D., AND
FLOYD W. McRAE, M. D.

### *ORIGINAL COMMUNICATIONS.*

### THE SURGICAL TREATMENT OF PUERPERAL PERITONITIS.*

BY
W. E. B. DAVIS, M. D.,

President of the Tri-State Medical Society of Alabama, Georgia and Tennessee; Secretary of the Southern Surgical and Gynecological Association, etc.
ROME, GEORGIA.

Puerperal fever is no longer regarded as a distinct affection, a disease *sui generis*, but is looked upon as a septic fever, resulting from infection during the puerperal state. The relation of micro-organisms to puerperal infection has been well established, and is generally accepted. Peritonitis from puerperal infection requires the same consideration as septic peritonitis from other causes.

There are many cases of puerperal fever which produce death before the peritoneum becomes involved, the infection being so intense and profound that the patient succumbs before

*Abstract of a paper read before the Georgia Medical Association, April 21, 1892.

there are any local manifestations of the disease. The extension of the infection from puerperal sepsis may be through the lymphatics or veins, or it may extend up through the tubes. In the rapid form of infection, the poison is carried directly into the circulation. Where there is a general peritonitis, it has usually resulted by infection through the lymphatics, and hence general peritonitis from puerperal sepsis cannot occur without general infection.

Puerperal septicæmia, as pointed out by Charpentier may be divided into two great classes; one in which nearly all cases die, and the other in which nearly all cases get well, if properly treated. In the first class are those cases in which the whole system is rapidly infected before there is any local evidence of inflammation, and under the same heading are those in which there is a general peritonitis with general infection. Under the second class may be placed those cases of mild, general infection with localized inflammatory troubles. In the last class, where the disease is limited to the pelvis, we have a fertile field for the surgeon, and much good has already been accomplished. There are cases that are operated on several weeks after delivery, and not cases of so-called puerperal fever which kills within four to ten days. A peritonitis that does not kill within a week or ten days may be regarded as localized, and such cases offer a chance for recovery if surgical procedures are adopted. There are many of these cases with pelvic abscesses, with septic infection, where the patient is so exhausted that nothing can be done more than to evacuate the pus, wash out the abscess and drain, so as to give the patient time to sufficiently recover to have the ovaries and tubes removed, which are the cause of the disease, and which make it impossible for the patient to be entirely well until they are extirpated. It may be said, however, that often after the evacuation of an abscess, the appendages are so much relieved that it does not become necessary to extirpate them, they having been drained through the abscess, just as an appendicitis is frequently cured by a perityphlitic abscess being drained. As pointed out, the system is often infected without any local manifestation whatever, and hence, no operation could offer relief. He has seen the abdomen opened in a case of this kind in which there was no local manifestation from the sepsis, except a moderately enlarged uterus. He had been called in consultation, by Dr. Copeland, of Birmingham, in such a case. The woman had been confined two weeks previously, and had a temperature from 102 to 104, which had begun soon after delivery. No local trouble could be found. The uterus had been curetted, but this gave no relief. He agreed with the doctor that they might find something by opening the abdomen, but nothing was found. The woman was suffering from pure septicæmia from puerperal infection. He would not advise an operation in a similar case ; for puerperal fevers that do not show some local trouble cannot be benefited by an operation.

Severe inflammations are usually made worse by pregnancy, and it is not an unusual thing where a woman has suffered very much with pelvic pain during gestation, that she will have fever after delivery, which is due to an old salpingitis. Should there be pus in a tube, the tube is liable to be ruptured and the pus emptied into the abdominal cavity, causing a general peritonitis, unless an operation is done very promptly, and it is not usual for the symptoms to be clear enough to warrant the surgeon in operating in time to save life. Perhaps when more attention is given to this particular complication of pregnancy, the surgeon may be called in at a time when an operation will be decided upon earlier than has been the practice heretofore. Cases of general, puerperal peritonitis have been operated on by a number of surgeons, but the results have been very unpromising. These cases offer but little from surgical procedures.

When quarts and gallons of pus are reported as having been removed from the general peritoneal cavity and recovery followed, he believes that the pus has usually resulted from a local collection, which has ruptured into the general cavity, and the operation has been done before sufficient time had elapsed for this amount of pus to result from the septic, inflammatory process in the general cavity. It is easy to understand how a gallon of pus, which has been shut off from the general cavity by inflammatory exudations and adhesions, and which has only recently ruptured into the peritoneal cavity can be removed and recovery follow; and it is not difficult to comprehend how this condition might be mistaken for acute, general, suppurative peritonitis, with a gallon or a quart of pus as a result in the cavity; as the pus, by its irritating properties, will produce an inflammation which would be misleading, but the condition is quite different from what would be had if the pus had been the result of a general inflammation. True, there would be many grave symptoms, but not that extensive, local inflammation and fatal toxemia, which would result from a peritonitis which had existed long enough to give rise to so large a quantity of free pus in the cavity.

Pathological and clinical study, combined with bacteriological and experimental research, has demonstrated conclusively that where a general peritonitis has become purulent there have resulted such destructive local effects and so profound a general infection that the condition must be considered fatal.

A suppurative or purulent peritonitis is usually localized; in other words, a septic inflammation that goes on to pus formation, is, as a rule, a localized inflammation, and the pus is limited by inflammatory adhesions, so as to protect the general cavity from infection. A septic inflammation that involves the general peritoneal cavity will generally produce death before pus has had time to form, and hence, septic inflammation, as a rule, must be localized, in order for the patient to survive long enough for pus formation.

In mild cases of infection following delivery, it is best to wash out the uterus every four hours with sterilized water, and if this does not reduce the temperature and relieve the symptoms, the curette should be used. Where this fails to give relief, and no local inflammation can be made out, it is well to purge the patient, that the system may be freed from sepsis as much as possible. Where the abdomen is tender, it is always better to give purgatives. The symptoms will usually be improved after this treatment, unless there be present a general septic peritonotis. The pain of a localized inflammation will be ameliorated by free purgation. A woman with a pelvic abscess, or a large pus tube, will feel easier after the bowels have been made to move six or eight times. In those cases where we have tympanites accompanying a local inflammation of the abdomen, and where it is doubtful whether or not there is present a general peritonitis, purgation relieves the tympanites, and enables one to locate the disease, as all the symptoms simulating a general peritonitis will have been relieved by the purgative, and the tenderness will then be confined to the area of localized inflammation. So, aside from its curative value, the purgative treatment of peritonits is a most valuable diagnostic measure. It enables the physician to say whether there is a general or localized inflammation or not, although it is not possible to say, in all cases, whether or not there is pus. Of course, if there should be a general peritonitis revealed by failure of purgation to relieve inflammation, generally there would be very little hope of relief by surgical procedures, for the inflammation by that time would have become so general and so intense, and the system so infected that the disease would be incurable.

As stated, localized peritonitis, with pus formation, is the form of the disease that is amenable to surgical treatment. He has operated on cases, where there seemed to be no hope of recovery, with favorable results. He has seen cases where the pulse was 135 get well after an operation for the relief of this condition. In 1889 he did an operation on Mrs. M., of Green Pond, Ala., for Dr. Payne, six weeks after her delivery, and removed nearly a gallon of stinking pus. When he saw the woman she was nearly dead. The abscess was opened, washed out and thoroughly drained. The woman was so weak that it was necessary to support her by rectal alimentation for ten days. Soon after the operation her pulse ran up from 150 to 160 degrees. She is now entirely well and has no trouble whatever from her ovaries and tubes.

In December, 1891, he operated on a patient for Dr. Sexton, of Birmingham, Ala., whose pulse was over 130, and who looked to be in a dying condition. More than half a gallon of pus was removed from this case. One-half of her abdomen, the left side, was an abscess. She was infected by an abortion. She had an uninterrupted recovery and is now well.

Frequently a fever from puerperal infection may continue for weeks where there is no pus formation, and in which the ovaries are greatly enlarged and the broad ligament thickened with occlusion of the tubes. He has operated on a case where the tissues were so soft that the ligatures would cut through, and it was necessary to take up the vessels and ligate them separately. An ovary in this condition is as septic as if it contained pus. Frequently the masses felt in the pelvis following delivery or abortion are due to pelvic peritonitis, with adhesions to the intestine and omentum, and when an operation is made to evacuate pus, there will often be found, in the place of pus, this condition present, but where fever is kept up, and the patient's condition shows sepsis, it is wiser to open the abdomen, break up these adhesions and remove the diseased appendages, which are keeping up the infection.

